# Alpha‐Synuclein is Involved in DYT1 Dystonia Striatal Synaptic Dysfunction

**DOI:** 10.1002/mds.29024

**Published:** 2022-04-14

**Authors:** Giulia Ponterio, Gaia Faustini, Ilham El Atiallah, Giuseppe Sciamanna, Maria Meringolo, Annalisa Tassone, Paola Imbriani, Silvia Cerri, Giuseppina Martella, Paola Bonsi, Arianna Bellucci, Antonio Pisani

**Affiliations:** ^1^ Laboratory of Neurophysiology and Plasticity IRCCS Fondazione Santa Lucia Rome Italy; ^2^ Division of Pharmacology, Department of Molecular and Translational Medicine University of Brescia Brescia Italy; ^3^ Department of Systems Medicine University of Rome Tor Vergata Rome Italy; ^4^ UniCamillus‐Saint Camillus International University of Health Sciences Rome Italy; ^5^ IRCCS Fondazione Mondino Pavia Italy; ^6^ Department of Brain and Behavioral Sciences University of Pavia Pavia Italy

**Keywords:** α‐synuclein, dystonia, striatum, SNAREs, asynchronous glutamate release, synaptic vesicle turnover

## Abstract

**Background:**

The neuronal protein alpha‐synuclein (α‐Syn) is crucially involved in Parkinson's disease pathophysiology. Intriguingly, torsinA (TA), the protein causative of DYT1 dystonia, has been found to accumulate in Lewy bodies and to interact with α‐Syn. Both proteins act as molecular chaperones and control synaptic machinery. Despite such evidence, the role of α‐Syn in dystonia has never been investigated.

**Objective:**

We explored whether α‐Syn and N‐ethylmaleimide sensitive fusion attachment protein receptor proteins (SNAREs), that are known to be modulated by α‐Syn, may be involved in DYT1 dystonia synaptic dysfunction.

**Methods:**

We used electrophysiological and biochemical techniques to study synaptic alterations in the dorsal striatum of the Tor1a^+^/^Δgag^ mouse model of DYT1 dystonia.

**Results:**

In the Tor1a^+/Δgag^ DYT1 mutant mice, we found a significant reduction of α‐Syn levels in whole striata, mainly involving glutamatergic corticostriatal terminals. Strikingly, the striatal levels of the vesicular SNARE VAMP‐2, a direct α‐Syn interactor, and of the transmembrane SNARE synaptosome‐associated protein 23 (SNAP‐23), that promotes glutamate synaptic vesicles release, were markedly decreased in mutant mice. Moreover, we detected an impairment of miniature glutamatergic postsynaptic currents (mEPSCs) recorded from striatal spiny neurons, in parallel with a decreased asynchronous release obtained by measuring quantal EPSCs (qEPSCs), which highlight a robust alteration in release probability. Finally, we also observed a significant reduction of TA striatal expression in α‐Syn null mice.

**Conclusions:**

Our data demonstrate an unprecedented relationship between TA and α‐Syn, and reveal that α‐Syn and SNAREs alterations characterize the synaptic dysfunction underlying DYT1 dystonia. © 2022 The Authors. *Movement Disorders* published by Wiley Periodicals LLC on behalf of International Parkinson Movement Disorder Society.

Impairment of the synaptic vesicles machinery and neurotransmission is a characteristic feature of different movement disorders, including Parkinson's disease (PD), dystonia, and parkinsonism with dystonia.^1^, ^2^

Alpha‐synuclein (α‐Syn), a synaptic enriched protein member of the synucleins family, participates in the neuropathophysiology of PD.^3^, ^4^ Besides PD, its pathological aggregates characterize a wider group of neurodegenerative disorders defined as synucleinopathies.^3^, ^4^ In humans, α‐Syn is encoded by the *SNCA* gene, located on chromosome 4q21. The main SNCA transcript gives rise to the production of a protein of 140 amino acids, which is ubiquitously expressed in the peripheral and central nervous system.^4^

Although α‐Syn functions are not entirely disclosed, it is known to play a role in maintaining the recycling pool of synaptic vesicles and modulating the assembly of soluble N‐ethylmaleimide‐sensitive factor attachment protein receptor (SNARE) complex.^5^, ^6^ In particular, α‐Syn acts as a chaperone to promote SNARE complex assembly and to limit the trafficking and recycling of synaptic vesicles, thus controlling neurotransmitter release also by direct binding to vesicle‐associated membrane protein‐2 (VAMP‐2/synaptobrevin‐2).^6^, ^7^ Conversely, the pathological deposition of α‐Syn in insoluble aggregates at synaptic terminals affects SNARE proteins (SNAREs) distribution in the brain of PD patients and in experimental synucleinopathy models exhibiting neurotransmitter release failure.^8^, ^9^ These findings are of particular interest in the context of PD and Lewy body (LB) dementia, as in the brains of the patients affected by these disorders the deposition of α‐Syn aggregates at synaptic sites is several orders of magnitude higher than the amount of the protein composing LB.^10^ Despite compelling evidence supporting the existence of possible overlapping mechanisms in PD and dystonia,^11^ the role of α‐Syn and SNAREs in the latter has never been investigated.

Early‐onset generalized torsion DYT1 dystonia (DYT1) is an autosomal dominant movement disorder caused by a GAG deletion in the *TOR1A* gene coding for torsinA (TA).^12^ Loss of the reciprocal modulation between the dopaminergic and cholinergic systems and synaptic plasticity imbalance point to synaptic dysfunction as a major pathophysiological alteration of DYT1 dystonia.^13^, ^14^, ^15^ TA is a member of the AAA+ superfamily of ATPases, which typically act as chaperones in the endoplasmic reticulum (ER).^16^ However, the interaction between TA and snapin supports the hypothesis that TA may influence synaptic vesicles dynamics in neurons.^17^, ^18^ Indeed, ΔE‐TA overexpression affects vesicle exocytosis, thus resulting in the accumulation of the calcium (Ca^2+^) sensor synaptotagmin I (Syt I) on the plasma membrane through a mechanism that involves snapin regulation.^17^ In this way, TA acts as a chaperone at the synapse level affecting synaptic vesicles turnover and neurotransmitter release.^19^ Intriguingly, TA has been found to accumulate in LB, where it interacts with α‐Syn.^20^ Moreover, a recent study has shown that dystonia‐related genes may converge in common pathways linked to α‐Syn and synaptic signaling.^21^ Consistently, α‐Syn null mice exhibit a decrease in striatal dopamine release as well as in the expression of some synaptic markers in the striatum, such as Syt and the dopamine transporter (DAT).^22^ α‐Syn and TA can both modulate DAT trafficking^23^, ^24^, ^25^ and affect corticostriatal plasticity.^14^, ^26^, ^27^ The two proteins are detectable in striatal synapses where α‐Syn is mostly localized at glutamatergic terminals and controls the mobilization of glutamate from reserve pools.^28^, ^29^, ^30^, ^31^ Finally, TA can affect synaptic vesicle recycling analogously to α‐Syn.^17^, ^32^, ^33^, ^34^, ^35^ Together, these findings suggest that both TA and α‐Syn play a major role in the control of synaptic homeostasis.^36^, ^37^, ^38^

Here, we investigated the possible occurrence of alterations in α‐Syn and SNAREs levels in association with functional changes in the striatum of the Tor1a^+/Δgag^ knock‐in DYT1 mouse model. Our findings reveal that Tor1a^+/Δgag^ mice exhibit a specific reduction of α‐Syn levels in glutamatergic striatal terminals in association with an imbalance of synaptic proteins related to the SNARE complex. In parallel, we observed a remarkable decrease of miniature and quantal excitatory postsynaptic currents (mEPSC and qEPSC, respectively) recorded from striatal spiny projection neurons (SPNs), in the absence of alterations in GABAergic currents, indicating a significant impairment in release probability. These findings suggest that alterations in α‐Syn expression and SNAREs may cause vesicle recycling alterations, with an ensuing impact on synaptic activity and plasticity.

## Results

### Striatal Levels of α‐Syn are Reduced in Tor1a^+/Δgag^ DYT1 Mouse Model

**FIG 1:**
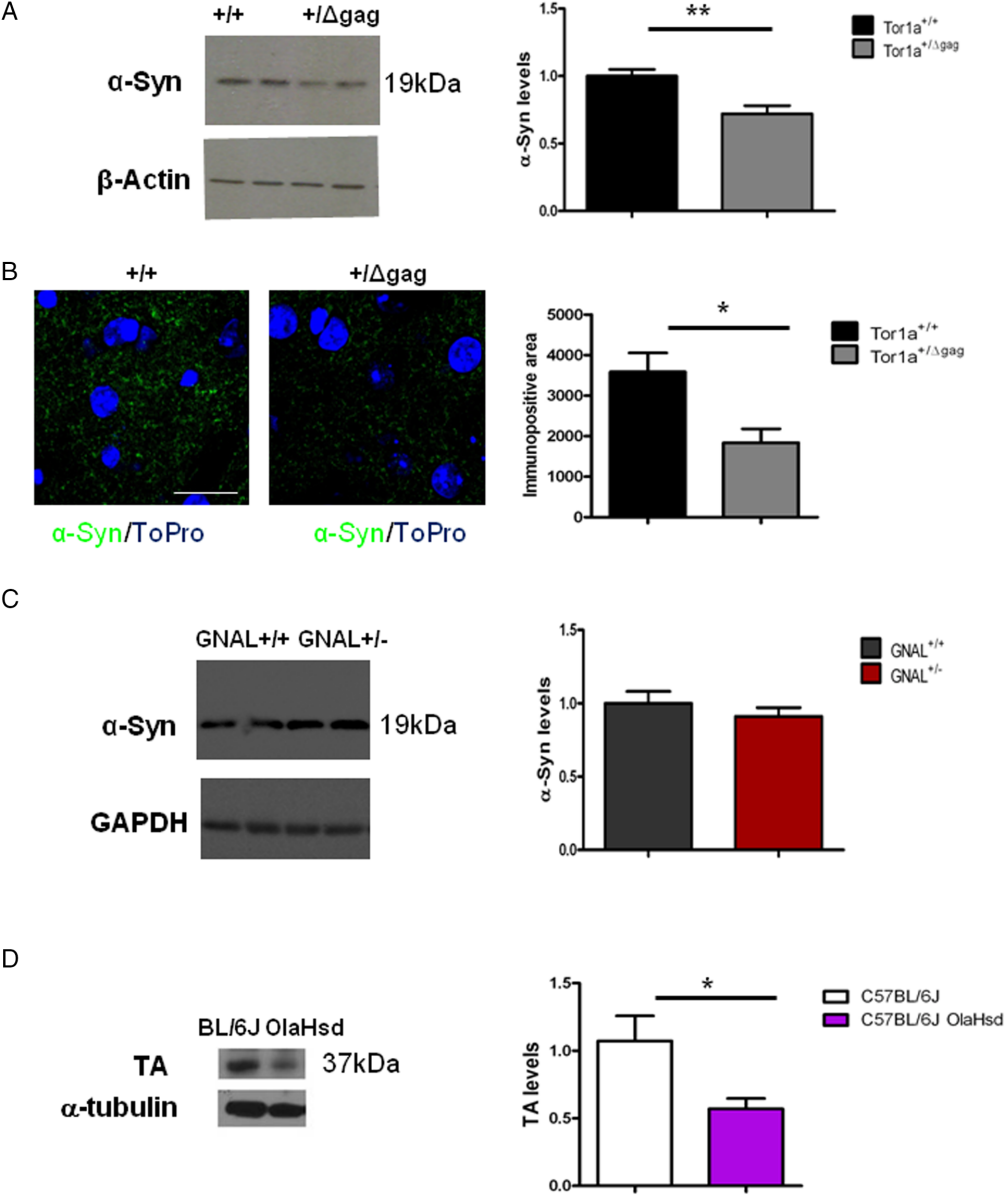
Striatal levels of alpha‐synuclein (α‐Syn) are reduced in Tor1a^+/Δgag^ DYT1 dystonia mouse model. (**A**) Representative Western Blot (WB) showing α‐Syn protein level reduction in the dorsal striata from Tor1a^+/Δgag^ mice. The graph shows the quantitative analysis of α‐Syn levels normalized to Tor1a^+/+^ mice. The amount of α‐Syn was quantified relatively to β‐actin. Data are presented as mean ± SEM (Tor1a^+/+^ = 1 ± 0.05, N = 14; Tor1a^+/Δgag^ = 0.72 ± 0.06, N = 12; **P < 0.01). (**B**) Representative confocal images showing a reduction in the α‐Syn fluorescence signal in the dorsal striatum of Tor1a^+/Δgag^ and *Tor1a* ^+/+^ mice (Tor1a^+/+^ = 3546 ± 489 μm^2^, N = 6; Tor1a^+/Δgag^ = 1837 ± 341 μm^2^, N = 5; *P < 0.05). Scale bar = 20 μm. (**C**) Representative WB showing α‐Syn protein level unchanged in the dorsal striata from GNAL^+/−^ rat. The graph shows the quantitative analysis of α‐Syn levels normalized to *GNAL* ^*+/+*^ rat. The amount of α‐Syn was quantified relatively to GAPDH levels. Data are presented as mean ± SEM (GNAL^+/+^ = 1 ± 0.08, N = 9; GNAL^+/−^ = 0.91 ± 0.06, N = 9; not significant [NS]) (**D**) Representative WB showing torsinA (TA) protein level reduction in the dorsal striata from α‐Syn null mice. The graph shows the quantitative analysis of TA. The amount of ΤΑ was quantified relatively to α‐tubulin (C57BL/6J = 1.07 ± 0.19, N = 4; C57BL/6JOlaHsd = 0.57 ± 0.08, N = 4; *P < 0.05). [Color figure can be viewed at wileyonlinelibrary.com]

The loss of the reciprocal modulation between the dopaminergic and cholinergic systems and corticostriatal plasticity imbalance suggest that synaptic dysfunction contributes to the pathophysiology of DYT1 dystonia.^13^, ^14^ Western Blot studies and confocal imaging were performed to analyze the levels of α‐Syn in the dorsal striatum of Tor1a^+/Δgag^ DYT1 mice. In striatal lysates from mutant mice, we observed significantly reduced levels of α‐Syn protein compared to control samples (Fig. 1A; ** *P* < 0.01). In addition, confocal analysis showed a significant reduction of α‐Syn‐positive signal in mutant mice striatum compared to control (Fig. 1B; *P < 0.05). Interestingly, this downregulation was peculiar for DYT1, since the striatal levels of α‐Syn were unchanged in a different dystonia model, the GNAL (DYT25) rat model (Fig. 1C; *P* > 0.05). DYT1 mice exhibit a decrease of TA levels of approximately 50% with respect to wild‐type littermates, suggesting that the Δgag is a loss‐of‐function mutation.^38^, ^39^ To evaluate whether α‐Syn could affect TA protein levels, we also assessed striatal TA levels in α‐Syn null mice. Surprisingly, we found a significant reduction (47%) of striatal TA level in α‐Syn null mice compared to controls (Fig. 1D; *P < 0.05), suggesting the existence of a reciprocal modulatory interaction between these two proteins.

### Impaired Protein Expression of SNAREs Complex in Tor1a^+/Δgag^ Mice

**FIG 2:**
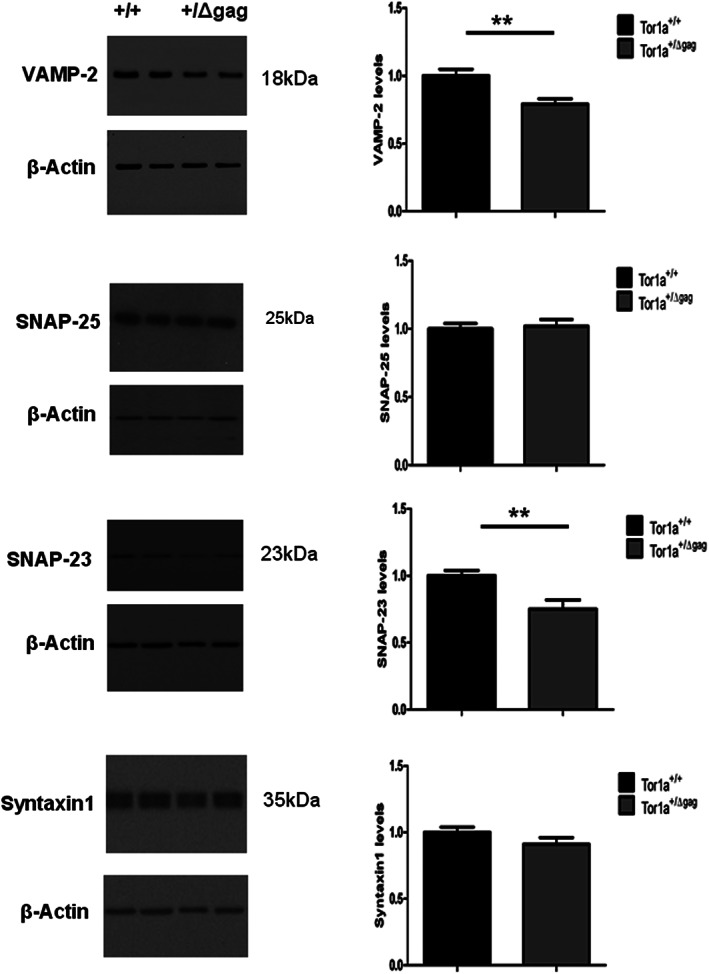
Striatal levels of soluble N‐ethylmaleimide‐sensitive factor attachment protein receptor (SNARE) proteins in Tor1a^+/Δgag^ DYT1 dystonia mouse model. Representative Western Blot (WB) showing SNARE proteins level in the dorsal striata of Tor1a^+/Δgag^ and *Tor1a* ^+/+^ mice. The graphs show the quantitative analysis of vesicle‐associated membrane protein‐2 (VAMP‐2), synaptosome‐associated protein 25 (SNAP‐25), SNAP‐23, and syntaxin 1 normalized to *Tor1a* ^+/+^ mice. The amount of proteins were quantified relatively to β‐actin. Data are presented as mean ± SEM (VAMP‐2: Tor1a^+/+^ = 1 ± 0.05, N = 11; Tor1a^+/Δgag^ = 0.79 ± 0.04, N = 11; **P < 0.01; SNAP‐25: Tor1a^+/+^ = 1 ± 0.04, N = 15; Tor1a^+/Δgag^ = 1.02 ± 0.05, N = 11; not significant [NS]; SNAP‐23: Tor1a^+/+^ = 1 ± 0.04, N = 13; Tor1a^+/Δgag^ = 0.75 ± 0.07, N = 14; **P < 0.01; syntaxin 1: Tor1a^+/+^ = 1 ± 0.04, N = 11; Tor1a^+/Δgag^ = 0.91 ± 0.05, N = 10; NS).

The SNARE complex mediates the fusion between synaptic vesicles and the presynaptic terminals. It consists of a number of proteins including the vesicle‐associated SNAREs (v‐SNAREs) VAMP‐2 and the target cell‐associated SNAREs (t‐SNAREs) syntaxin I and synaptosome‐associated protein 25 kD (SNAP‐25) or homologs. SNARE complex formation is maintained by canonical chaperones but also by non‐classical chaperones such as α‐Syn.^40^ Since α‐Syn may promote SNARE complex assembly through direct binding to VAMP‐2^5^, ^7^ we quantified the expression of syntaxin‐1, VAMP‐2, SNAP‐25, and its ubiquitously expressed homolog SNAP‐23 in striatal lysates. We found that the levels of VAMP‐2 were significantly reduced in the lysates from Tor1a^+/Δgag^ mice when compared to Tor1a^+/+^ samples (Fig. 2; ** *P* < 0.01). In addition, in the lysates from Tor1a^+/Δgag^ mice we also observed a significant reduction of SNAP‐23 (Fig. 2; **P < 0.01) when compared to Tor1a^+/+^ samples, although the (t‐SNAREs) syntaxin‐1 and SNAP‐25 were unchanged (Fig. 2; *P* > 0.05).

### α‐Syn Co‐localizes with VGLUT‐1 in Corticostriatal Glutamatergic Terminals

**FIG 3:**
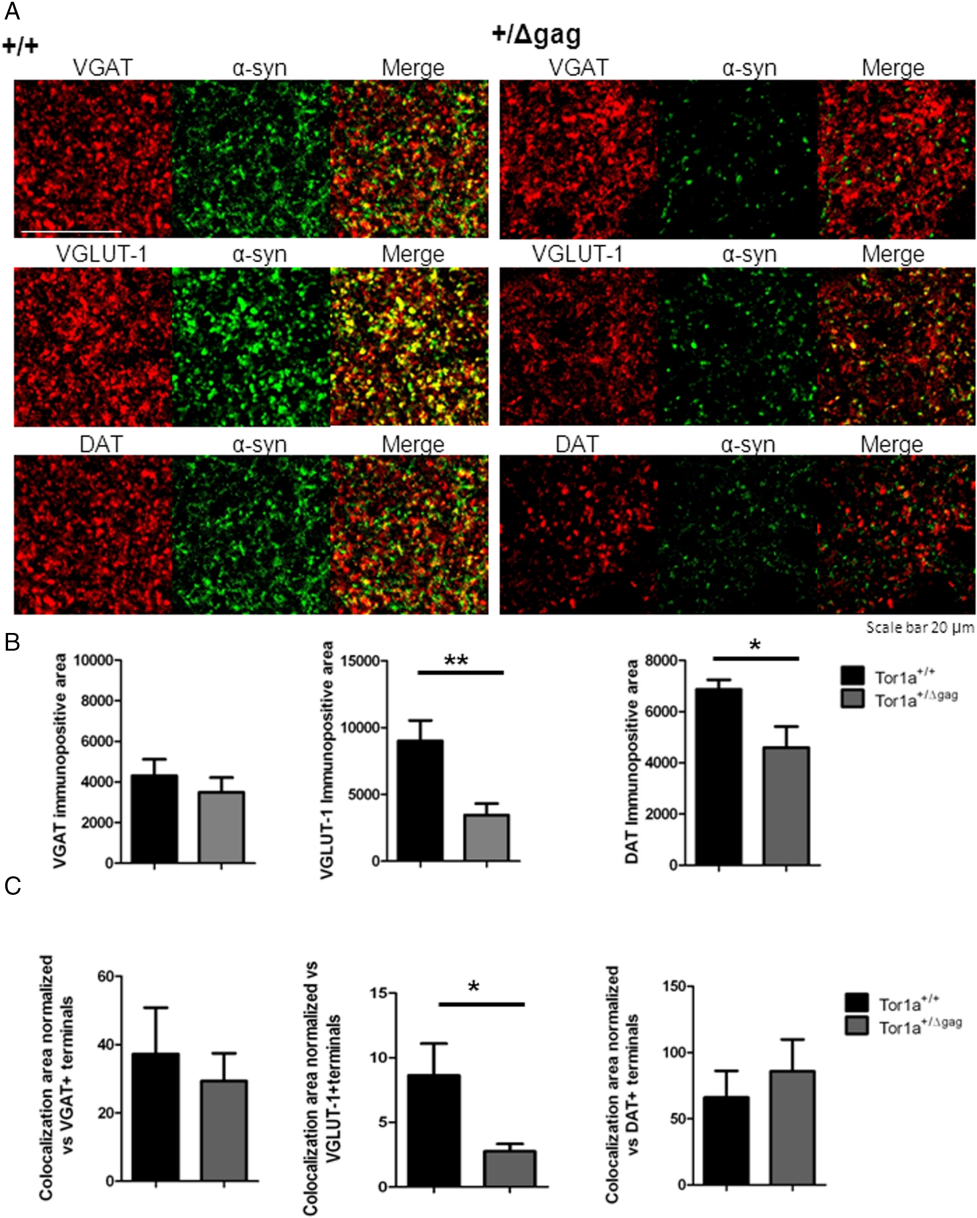
Analysis of alpha‐synuclein (α‐Syn) co‐localization with vesicular GABA transporter (VGAT), vesicular glutamate transporter‐1 (VGLUT‐1), and dopamine transporter (DAT) in the striatum of Tor1a^+/Δgag^ and *Tor1a* ^+/+^ mice. (**A**) Representative double‐immunofluorescence confocal images show the co‐labeling of α‐Syn with GABAergic marker, glutamatergic marker, and dopaminergic marker: VGAT, VGLUT‐1, and DAT, respectively. Scale bar = 20 μm. (**B**) Graphs show the immunopositive area of VGAT, VGLUT‐1, and DAT (VGAT: Tor1a^+/+^ = 4310 ± 807 μm^2^, N = 6; Tor1a^+/Δgag^ = 3497 ± 723 μm^2^, N = 7; VGLUT‐1: Tor1a^+/+^ = 9008 ± 1531 μm^2^, N = 6; Tor1a^+/Δgag^ = 3454 ± 856 μm^2^, N = 7; **P < 0.01; DAT: Tor1a^+/+^ = 6861 ± 379 μm^2^, N = 6; Tor1a^+/Δgag^ = 4593 ± 816 μm^2^, N = 7; *P < 0.05). (**C**) Histograms show the co‐localization area between α‐Syn and the respective synaptic marker VGAT, VGLUT‐1, and DAT terminals normalized versus the overall amount of the VGAT‐, VGLUT‐1‐, or DAT‐immunopositive area, respectively (VGAT: Tor1a^+/+^ = 37.25 ± 13.53 μm^2^, N = 6; Tor1a^+/Δgag^ = 29.28 ± 8.16 μm^2^, N = 7; not significant [NS]; VGLUT‐1: Tor1a^+/+^ = 8.62 ± 2.47 μm^2^, N = 6; Tor1a^+/Δgag^ = 2.75 ± 5.87 μm^2^, N = 7; *P < 0.05; DAT: Tor1a^+/+^ = 65.97 ± 20.1 μm^2^, N = 6; Tor1a^+/Δgag^ = 85.68 ± 24.2 μm^2^, N = 7; NS). [Color figure can be viewed at wileyonlinelibrary.com]

In the striatum, α‐Syn is most abundant in excitatory when compared to inhibitory synapses and co‐localizes mainly with vesicular glutamate transporter‐1 (VGLUT‐1), and, to a lesser extent, with VGLUT‐2.^29^, ^41^, ^42^ Thus, in order to evaluate possible changes of striatal dopaminergic, glutamatergic, and GABAergic synaptic terminals, we measured the areas immunopositive for the specific markers DAT, VGLUT‐1, and vesicular GABA transporter (VGAT), respectively. (Fig. 3A). We found a significant reduction of DAT‐ and VGLUT‐1‐immunopositive areas (Fig. 3B; *P < 0.05 and **P < 0.01, respectively), while the VGAT‐positive area was unchanged (Fig. 3B; *P* > 0.05). Then, we performed a co‐localization analysis of the areas positive for both α‐Syn and the specific immunolabeling for the different synaptic markers (Fig. 3A). In particular, we assessed the co‐localization rate, which represents the α‐Syn immunopositive signal (in pixels) overlapping with the immunopositive signal of each of the specific markers (DAT, VGLUT‐1, and VGAT). This co‐localization rate was then normalized versus the overall area of immunopositivity of each of the assessed markers in order to estimate the amount of α‐Syn localized in DAT‐, VGLUT‐1‐, or VGAT‐positive terminals. Interestingly, the co‐localization analysis showed a significant decrease only in the amount of α‐Syn localizing within VGLUT‐1‐immunopositive corticostriatal terminals in Tor1a^+/Δgag^ mice when compared to Tor1a^+/+^ animals (Fig. 3C; *P < 0.05), while no changes were detected in the α‐Syn within VGAT‐ and DAT‐positive terminals. This supports the observation that mutant TA‐associated striatal α‐Syn decrease mainly involves glutamatergic terminals.

### Glutamatergic mEPSC are Altered in Tor1a^+/Δgag^ Mice

**FIG 4:**
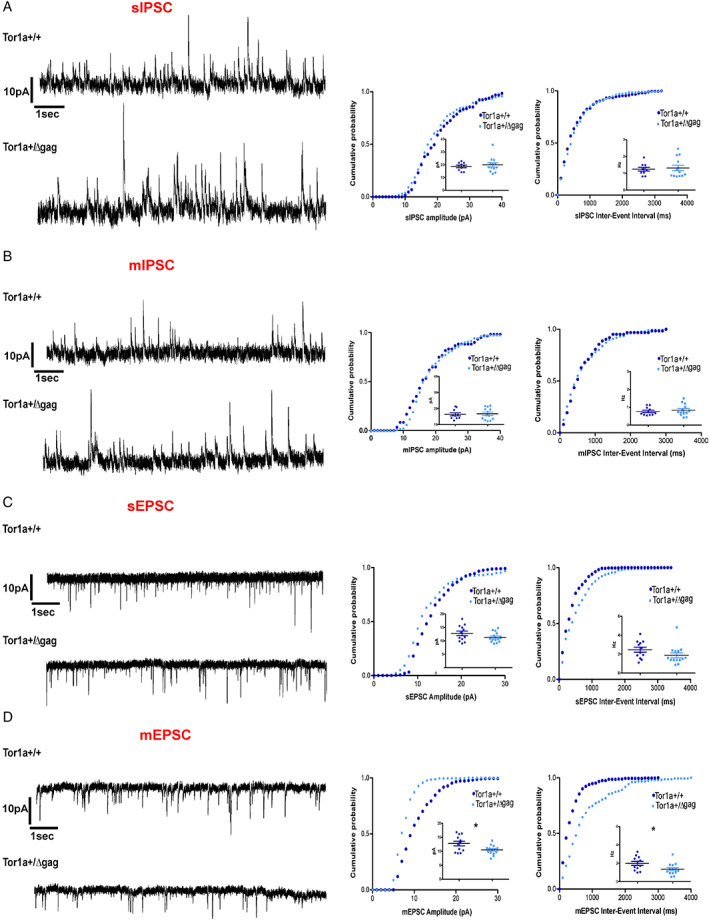
Miniature excitatory (glutamatergic) postsynaptic currents (mEPSCs) were altered in *Tor1a* ^+/Δgag^ mice. (**A**) Representative cumulative distribution curves and spontaneous inhibitory postsynaptic currents (sIPSCs) recordings in MK‐801 and CNQX from spiny projection neurons (SPNs) of Tor1a^+/+^ and Tor1a^+/Δgag^ mice. HP: +10 mV. The summary plots show no significant difference in sIPSC frequency (Tor1a^+/+^ 1.24 ± 0.10 Hz, n = 10; Tor1a^+/Δgag^ 1.31 ± 0.16 Hz, n = 13; not significant [NS]) and amplitude (Tor1a^+/+^ 18.70 ± 0.97 pA, n = 10; Tor1a^+/Δgag^ 19.94 ± 1.6 pA, n = 13; NS). (**B**) Representative cumulative distributions curves and miniature inhibitory postsynaptic currents (mIPSCs) recordings in MK‐801 and CNQX plus TTX from Tor1a^+/+^ and Tor1a^+/Δgag^ SPNs. The summary plots show no significant difference in mIPSCs frequency (Tor1a^+/+^ 0.77 ± 0.07 Hz, n = 10; Tor1a^+/Δgag^ 0.83 ± 0.09 Hz, n = 13; NS) and amplitude (Tor1a^+/+^ 16.42 ± 0.96 pA, n = 10; Tor1a^+/Δgag^ 16.75 ± 1.0 pA, n = 13; NS). (**C**) Representative cumulative distribution curves and spontaneous excitatory postsynaptic currents (sEPSCs) recordings in PTX from SPNs of Tor1a^+/+^ and Tor1a^+/Δgag^ mice. HP: –70 mV. The summary plots show no significant difference in sEPSC frequency (Tor1a^+/+^ 2.47 ± 0.26 Hz, n = 12; Tor1a^+/Δgag^ 1.88 ± 0.26 Hz, n = 14; NS) and amplitude (Tor1a^+/+^ 12.79 ± 0.85 pA, n = 12; Tor1a^+/Δgag^ 11.31 ± 0.50 pA, n = 14; NS). (**D**) Representative cumulative distribution curves and mEPSCs recordings in PTX plus TTX from Tor1a^+/+^ and Tor1a^+/Δgag^ SPNs. The summary plots show significant difference in mEPSCs frequency (Tor1a^+/+^ 1.97 ± 0.21 Hz, n = 12; Tor1a^+/Δgag^ 1.34 ± 0.17 Hz, n = 14; *P < 0.05) and amplitude (Tor1a^+/+^ 12.77 ± 0.83 Hz, n = 12; Tor1a^+/Δgag^ 10.48 ± 0.44 Hz, n = 14; *P < 0.05). [Color figure can be viewed at wileyonlinelibrary.com]

α‐Syn limits the trafficking and recycling of synaptic vesicles attenuating neurotransmitter release by its interaction with VAMP‐2.^7^, ^43^, ^44^ To explore potential differences in neurotransmitter release induced by a reduced expression of α‐Syn, VAMP‐2, and SNAP‐23, we performed whole‐cell patch‐clamp recording experiments to analyze spontaneous inhibitory (GABA‐mediated) and excitatory (glutamate‐mediated) postsynaptic currents (sIPSCs and sEPSCs, respectively) in SPNs from both Tor1a^+/+^ and Tor1a^+/Δgag^ mice. Then, we recorded the frequency and amplitude of miniature currents (mIPSC and mEPSC) to evaluate presynaptic vesicle release. GABAergic sIPSCs and mIPSCs were unchanged in Tor1a^+/Δgag^ with respect to Tor1a^+/+^ littermates (Fig. 4A,B; *P* > 0.05) in line with our recent observations.^45^ In addition, glutamatergic sEPSCs did not differ between genotypes (Fig. 4C; *P* > 0.05), as previously demonstrated.^14^ However, we found a significant decrease in the amplitude and frequency of mEPSCs recorded from Tor1a^+/Δgag^ mice when compared to wild‐type animals (Fig. 4D; *P < 0.05). No changes in kinetic properties were observed between genotypes (data not shown; decay time constant: Tor1a^+/+^ 8.18 ± 0.86 ms; Tor1a^+/Δgag^ 11.10 ± 1.52 ms, rise time: Tor1a^+/+^ 3.11 ± 0.19 ms; Tor1a^+/Δgag^ 3.50 ± 0.26 ms; *P* > 0.05). The reduction of mEPSC reflects an impairment of the vesicular glutamate content that is reminiscent of that observed in α‐Syn knockout (KO) mice.^31^ Of note, western blot analysis showed a down‐regulation of SNAP‐23 which, unlike SNAP‐25, is important for the functional regulation of glutamate receptors.^46^ Remarkably, these are pivotal for glutamatergic transmission that is significantly reduced in neurons from VGLUT‐1 KO mice where the loss of glutamate presynaptic loading and release impacts on synaptic vesicle cargoes turnover.^47^, ^48^ Our electrophysiological results also appear consistent with our present data showing a reduced α‐Syn expression specifically in the VGLUT‐1 terminals. Collectively, these data are supportive of the possible occurrence of an altered vesicle turnover, indicative of a dysfunctional presynaptic glutamatergic transmission in DYT1 mice.

### Downregulated Asynchronous Release in Tor1a^+/Δgag^ Mice

The synaptic membrane‐fusion machinery is controlled by Syt I, which acts as a calcium (Ca^2+^) sensor to regulate exocytosis during synchronous and asynchronous release.^49^, ^50^ Remarkably, while the synchronous release relies on the immediately releasable vesicles pool, asynchronous release can also involve recycling and reserve pools, which are regulated by α‐Syn.^33^, ^34^ In glutamatergic neurons, synchronous release requires SNAP‐25, while SNAP‐23 only supports asynchronous release.^51^ In addition, glutamatergic transmission is reduced in neurons from VGLUT‐1 KO mice, specifically in quantal size.^46^ Therefore, in order to corroborate our electrophysiological and biochemical data we investigated quantal‐like events (qEPSCs) evoked after corticostriatal stimulation in SPNs from Tor1a^+/+^ and Tor1a^+/Δgag^ mice following the replacement of extracellular Ca^2+^ with strontium (Sr^2+^).

**FIG 5:**
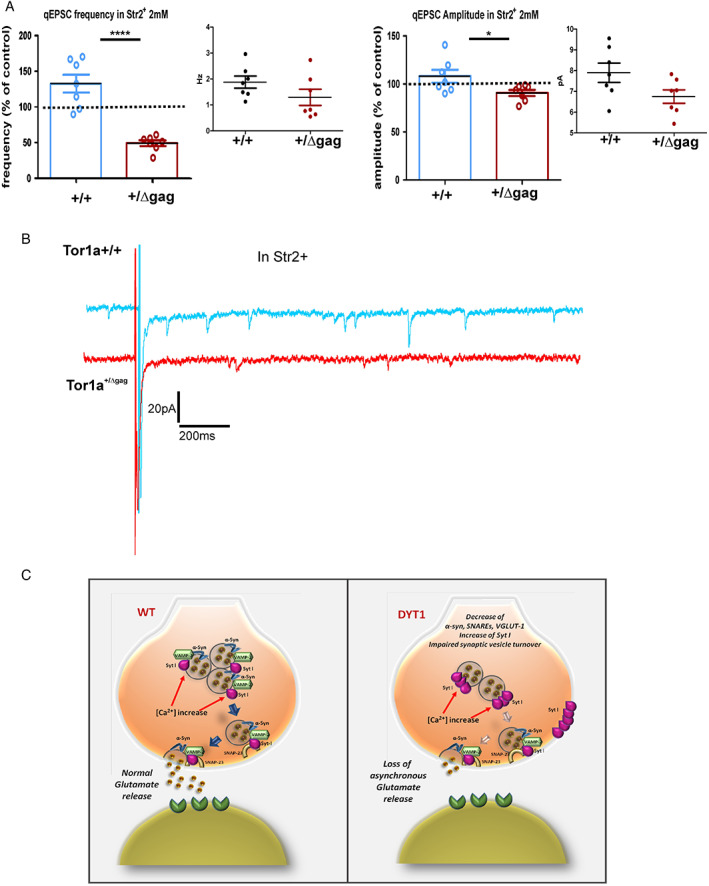
Downregulated glutamatergic asynchronous release in Tor1a^+/Δgag^ mice. (**A**) Top: Graphs summarize the change induced by strontium 2 mM in the frequency and amplitude of quantal excitatory postsynaptic currents (qEPSCs) evoked after corticostriatal stimulation in both strains. Values are expressed as percentages of control pre‐strontium (frequency Tor1a^+/+^ = 132.7 ± 12.6 Hz, n = 7, N = 7; Tor1a^+/Δgag^ = 49.19 ± 3.99 Hz, n = 7, N = 4; t‐test ****P < 0.0001; amplitude Tor1a^+/+^ = 108.1 ± 6.71 pA, n = 7, N = 7; Tor1a^+/Δgag^ = 90.68 ± 3.18 pA, n = 7, N = 4; t‐test *P < 0.05). Bottom: boxplots reporting frequency and amplitude changes of the qEPSC in both strains. (**B**) Representative traces showing the Sr^2+^‐induced asynchronous qEPSCs evoked after corticostriatal stimulation in Tor1a^+/+^ (n = 7) and Tor1a^+/Δgag^ (n = 7) mice. (**C**). Schematic model of synaptic dysfunction in DYT1 dystonia. Left: normal glutamatergic transmission. Right: loss of asynchronous glutamatergic release. In DYT1 the mutant torsinA (TA) induce an increase of synaptotagmin I (Syt I) level, a downregulation of alpha‐synuclein (α‐Syn), vesicle‐associated membrane protein‐2 (VAMP‐2), synaptosome‐associated protein 23 (SNAP‐23), and vesicular glutamate transporter‐1 (VGLUT‐1) with a consequent impairment of vesicles turnover and asynchronous glutamatergic release. [Color figure can be viewed at wileyonlinelibrary.com]

When Sr^2+^‐induced asynchronous release was recorded in Tor1a^+/ΔGAG^ SPNs, both the frequency and the amplitude of qEPSCs were significantly decreased compared to Tor1a^+/+^ neurons (Fig. 5A,B; ****P < 0.0001). Interestingly, our confocal analysis in the dorsal striatum from mutant mice showed a significant increase of the immunostaining of Syt I (Fig. S1A; **P < 0.01). Accordingly, the quantification Syt I levels revealed a significant increase in the Tor1a^+/Δgag^ striatum compared to Tor1a^+/+^ mice (Fig. S1B; *P < 0.05). The observation that Syt I governs the synaptic vesicle endocytosis time‐course by delaying the kinetics of vesicle retrieval in response to increasing Ca^2+^ levels^52^ supports the hypothesis that the increase of Syt I plays a role in the onset of asynchronous release deficits by affecting synaptic vesicles turnover. Indeed, the increase of Syt I may induce the formation of Syt I oligomers, which control asynchronous neurotransmitter release.^52^ Our findings are also consistent with previous evidence in DYT1 cell models showing that mutant TA overexpression promotes Syt I accumulation on the plasma membrane through the reduction of synaptic vesicle turnover.^17^, ^18^

Collectively, these data suggest that DΥΤ1 mutant mice exhibit a deficit in asynchronous glutamate release, which reflects a robust dysfunction of synaptic vesicle turnover.

## Methods

### Rodent Models and Experimental Design

Studies were carried out in adult (P60‐P90) mice and rats: knock‐in Tor1a^+/Δgag^ mice heterozygous for ΔE‐torsinA,^53^ in rats heterozygous for GNAL^54^ and α‐Syn null mice, carrying a spontaneous deletion of α‐Syn gene (Harlan Olac, Bicester, UK) and their respective wild‐type littermates (C57BL/6 for mice; Sprague Dawley for rats). Animal breeding and handling were performed in accordance with the guidelines for the use of animals in biomedical research provided by the European Union's directives and Italian laws (2010/63EU, D.lgs. 26/2014;86/609/CEE, D.Lgs 116/1992). Genotyping was performed as previously described.^55^, ^56^ Each observation was obtained from an independent biological sample. For electrophysiology, each cell was recorded from a different brain slice. All data were obtained from at least three animals in independent experiments. Biological replicates are represented with ‘N’ for number of animals and ‘n’ for number of cells.

### Slice Preparation and Electrophysiological Recordings

Mice were euthanized by cervical dislocation and the brain quickly removed from the skull. Electrophysiological patch‐clamp recordings were performed from individual SPN in striatal coronal slices, prepared as previously described.^45^ SPNs were visualized using standard infrared differential interference contrast (IR‐DIC) microscopy and identified based on their morphology and electrophysiological properties. Electrophysiological signals were detected using Multiclamp 700B and AxoPatch 200 amplifiers (Molecular Devices) using borosilicate glass pipettes pulled on a P‐97 Puller (Sutter Instruments). The electrodes were filled with cesium (Cs) + internal solution (in mM: 120 CsMeSO_3_, 15 CsCl, 8 NaCl, 10 TEA‐Cl, 10 HEPES, 0.2 EGTA, 2 Mg‐ATP, and 0.3 Na‐GTP; pH 7.3 adjusted with CsOH; 300 mOsm). For whole‐cell recordings of glutamatergic sEPSCs, SPNs were clamped at HP = –70 mV in the presence of the GABAA receptor antagonist PTX (50 μM). For GABAergic sIPSCs, were recorded at HP = +10 mV in MK801 (30 μM) and CNQX (10 μM) to block NMDA receptors (NMDARs) and AMPA receptors (AMPARs), respectively. Both mEPSCs and mIPSCs were measured by adding 1 μM TTX. Quantal events (qEPSC) were recorded after each stimulus (6 pulses delivered once every 10 s at 0.1 Hz for cortical stimulation) and external Ca^2+^ was replaced with Sr^2+^ (2 mM) as previously described.^13^, ^57^

### Western Blot

Western blot of striatal lysates was performed as previously described.^39^ Striata were homogenized in cold buffer: 50 mM Tris‐HCl pH 7.4, 150 mM NaCl, 1% Triton X‐100, 0.25% Na deoxycholate, 5 mM MgCl_2_, 0.1% SDS, 1 mM EDTA, and 1% protease inhibitor cocktail (Sigma‐Aldrich). Samples were sonicated and kept on ice for 1 hr. Then, crude lysates were centrifuged (13,000 rpm, 15 min, 4°C), the supernatant collected, and protein quantified with Bradford assay (Bio‐Rad). Protein extracts (5–10 μg) were loaded with NuPAGE LDS sample buffer (Invitrogen, Life Technologies) containing DTT. Samples were denatured (95°C, 5 min) and loaded onto 10%–12% SDS–PAGE gels. Gels were blotted onto 0.45‐lm polyvinylidene fluoride (PVDF) membranes. The following primary antibodies were used: rabbit (rb) anti‐α‐Syn 1:1000 (D37A6; Cell‐Signaling); rb anti‐TA 1:500 (ab34540; Abcam); mouse (ms) anti‐SNAP‐25 1:8000 (ab66066; Abcam); rb anti‐SNAP‐23 1:5000 (pab0057‐0; Covalab); ms anti‐VAMP‐2 1:10000 (104–211; Synaptic System); ms anti‐syntaxin 1 1:20000 (110–011; Synaptic System); rb anti‐Syt I 1:500 (ab131551; Abcam) overnight (ON) at 4°C; ms anti‐β‐actin (A5441; Sigma‐Aldrich); ms anti‐β‐tubulin (T4026; Sigma‐Aldrich); rb‐anti‐GAPDH (2118S; Cell‐Signaling) 30–60 min at room temperature (RT). Anti‐ms and anti‐rb horseradish peroxidase (HRP)‐conjugated secondary antibodies were used (GE Healthcare). Immunodetection was performed by ECL reagent (GE Healthcare) and the signal was detected using iBright CL1000 instrument (Thermo Fisher). Quantification was achieved by ImageJ software (NIH).

### Immunohistochemistry and Confocal Analysis

Mice were anaesthetized by intraperitoneal (i.p.) injection of chloral hydrate (400 mg/kg) (Sigma‐Aldrich) and perfused transcardially by using a 4% paraformaldehyde (PFA). After 4 hr of post‐fixation in 4% PFA, brains were incubated in a solution of phosphate‐buffered saline (PBS) with high salt concentration (NaOH 200 mM, NaH_2_PO_4_ 245 mM, NaCl 0.9%) containing 18% sucrose for at least 24 hr, then 25 μm coronal sections were cut with a cryostat (Leica Biosystems) and stored in 60% glycerol. After permeabilization in 20% methanol and 0.3% Triton X‐100 in PBS 0.1 M, the free floating sections were incubated for 1 hr at RT in blocking solution (2% v/v Normal Goat Serum (NGS), 3% w/v Bovine Serum Albumin (BSA), 0.3% Triton X‐100 in PBS 0.1 M), and then with the primary antibody diluted in blocking solution ON at 4°C. The following primary antibodies were used: ms anti‐α‐Syn 1:1000 (610,787; Beckton Dickson); rb anti‐VGAT (131‐002; SySy), rb anti‐VGLUT‐1 (135‐302; SySy), rat anti‐DAT (sc‐32258) goat‐anti‐DARP‐32 (AF6259; R&D System), and rb‐anti Syt I (ab131551; Abcam). The following day, sections were washed with 0.3% Triton X‐100 PBS 0.1 M and incubated with the fluorochrome‐conjugated secondary antibody in 0.3% Triton X‐100 PBS 0.1 M plus 1 mg/ml BSA for 1 hr at RT. The following secondary antibodies were used: goat anti‐rb AlexaFluor 488, goat anti‐ms Cy3, and anti‐rat Cy3 (Jackson Immunoresearch). After three washes in 0.3% Triton X‐100 PBS 0.1 M, sections were incubated for 2 hr at RT with the second primary antibody, followed by incubation for 1 hr at RT with the appropriate secondary antibody. Nuclei were stained with Topro‐3 (Thermo Fisher). Then, slices were mounted onto glass slides using Vectashield (Vector Laboratories) and analyzed by confocal microscopy. The slides were observed using an LSM 880 Zeiss confocal laser microscope with the laser set on λ = 405–488–543–633 nm and the height of the sections scanning = 1 μm. Images (512 × 512 pixels) were then reconstructed using ZEISS ZEN Imaging Software (Carl Zeiss).

### Image Analysis of Striatal Immunopositive Area

The acquisition parameters during confocal imaging were maintained constant for all the images acquired. The optical density of the striatal positive area from digitized images acquired by confocal microscopy were examined by a researcher blind to the experimental conditions using FIJI Software. Five sections from each mouse were analyzed by examining an average of 10 fields per section. The threshold setup for FIJI was fixed between 30 and 150. The area of co‐localization between α‐Syn‐immunolabeling and DAT‐, VGAT‐, or VGLUT‐1‐positive signal was quantified using Zen software (Carl Zeiss). The co‐localization rate was then normalized on the total DAT‐, VGAT‐, or VGLUT‐1‐positive area for each field, respectively, in order to estimate the percentage amount of α‐Syn‐immunoreactivity within each specific synaptic terminal.

### Quantification and Statistical Analysis

Data analysis was performed with MiniAnalysis 6.0, ImageJ (NIH), and Prism5.3 (GraphPad). Data are reported as mean ± SEM. Statistical significance was evaluated as indicated in the text, and two‐tailed unpaired or paired Student's test (t‐test) was used for two‐sample comparison. Normality tests were used to assess Gaussian distribution. Statistical tests were two‐tailed, the confidence interval was 95%, and the alpha‐level used to determine significance was set at *P* < 0.05.

## Discussion

The present results support the existence of an interplay between TA and α‐Syn in synaptic homeostasis that is particularly relevant for glutamate neurotransmission. In particular, our findings show that α‐Syn is downregulated in the striatum of mutant Tor1a^+/Δgag^ mice but not in a distinct dystonia model, the DYT25 GNAL rat model. A further clue to the α‐Syn–TA relationship is provided by our biochemical experiments on α‐Syn null mice, indicating a significant down‐regulation of TA. Our data also suggest that TA loss of function might alter synaptic machinery stability by inducing an increase in the Ca^2+^ sensor Syt I, and a decrease in α‐Syn and SNAREs thereby affecting the glutamate release process.

It has been shown that TA down‐regulation induces persistence of Syt I on plasma membrane suggesting that the DYT1 mutation compromises synaptic vesicle recycling.^17^, ^18^ Many studies point to a crucial role for Syt I in promoting the synchronous release coupling Ca^2+^ to SNARE‐mediated fusion mechanism, but also in suppressing the asynchronous release, especially upon oligomer formation.^58^, ^59^ We found high levels of Syt I in striatum from mutant mice, which may be supportive of Syt I oligomers, whose formation is relevant for the kinetics of synaptic vesicle recycling during asynchronous release,^52^ and that also appears in line with the increase of plasma membrane Syt I observed in DYT1 cell models.^17^ While Syt I deletion induces an increase in asynchronous neurotransmitter release, its increase can negatively impact on this process.^60^ Consistently, we found that in Tor1a^+/Δgag^ mice, Sr^2+^, which normally stimulates the asynchronous release with a consequent increase in events,^61^ reduced qEPSCs, thus confirming an impairment of asynchronous synaptic release. This finding, in parallel to the increase in Syt I, suggests that TA mutation impairs glutamatergic synaptic vesicles turnover, which in turn affects asynchronous glutamate release, a process governing the recovery of neuronal excitability following post‐spike hyperpolarization.^62^

We showed a significant reduction of the v‐SNAREs member VAMP‐2, which is the direct interactor of α‐Syn.^7^ Moreover, we also found a significant decrease of SNAP‐23, which, unlike SNAP‐25, is more important for the functional regulation of the glutamate receptors and in modulating asynchronous release.^46^, ^51^ Our present findings are in agreement with a proposed modulatory role of α‐Syn on glutamatergic synaptic activity, in line with evidence supporting it regulating presynaptic mobilization of reserve pools of vesicles at glutamatergic terminals.^31^, ^63^ Consistently, we found that in the Tor1a^+/Δgag^ mice α‐Syn was reduced at VGLUT‐1‐positive terminals, whereas GABAergic transmission was normal.

Our confocal imaging data demonstrate a diffuse reduction of VGLUT‐1 and DAT signals, in accordance with previous evidence on DYT1 experimental models.^64^, ^65^, ^66^ Indeed, Ip and co‐workers, showed that Tor1a^±^ mice exhibit a reduction of striatal DAT level as well as DAT binding decrease after sciatic nerve crush.^67^ This appears consistent with the fact that DAT is an α‐Syn interactor and that TA can affect DAT expression.^25^, ^67^ Of note, VGLUT‐1 plays a key role in controlling cargo protein recovery, including VAMP‐2, but not Syt I, and is essential for ensuring the quantal efficiency of glutamatergic transmission.^48^, ^68^ Therefore, the VGLUT‐1 reduction in DYT1 mice does not appear to contradict the observed Syt I accumulation. Interestingly, the relevance of VGLUT‐1 in cargo protein recovery may suggest that its decrease could also underlie the reduction of both α‐Syn and SNAREs. This notwithstanding, it has been shown that unlike other synaptic vesicle‐associated proteins such as SNAREs or synapsin, which rapidly recluster synaptic terminals co‐localizing with VGLUT‐1 in the post‐depolarization recovery phase, α‐Syn dissociates from synaptic vesicle membranes after their fusion and exhibits a different and slower recovery.^71^ Therefore, it appears unlikely that a VGLUT‐1 reduction‐associated lowering of cargo protein recovery could be the basis of the observed α‐Syn decrease at glutamatergic terminals, though we do not exclude the possibility that by affecting quantal synaptic efficiency^47^ it could blunt qEPSC.

Overall, our results highlight the existence of a strong relationship between TA, α‐Syn, and SNAREs in the control of glutamate release. In particular, they indicate that TA mutations affect striatal glutamatergic transmission mainly by impinging on asynchronous release. This phenomenon may very well be driven by the reduction of α‐Syn and SNAREs occurring in parallel to Syt I increase (Fig. 5C). Our findings further support the notion that different pathways may converge to cause basal ganglia synaptic abnormalities as a main determinant in the pathophysiology of dystonia.^21^, ^70^, ^71^ Furthermore, this evidence envisages a pivotal involvement of alterations of α‐Syn, SNAREs, and related synaptic vesicle‐associated protein in the molecular underpinnings of synaptic imbalance in DYT1 dystonia warranting further investigation.

## Financial Disclosures

A.P. is an employee at the University of Pavia, Pavia, Italy. P.B. and P.I. are employees at Fondazione Santa Lucia, Rome, Italy. A.B. is an employee at the University of Brescia, Brescia, Italy. The authors declare the absence of any potential conflicts of interest.

## Authors Roles

(1) Research Project: A. Conception, B. Organization, C. Execution; (2) Statistical Analysis: A. Design, B. Execution, C. Review and Critique; (3) Manuscript Preparation: A. Writing of the First Draft, B. Review and Critique.

G.P.: 1A, 1B, 1C, 2A, 2B, 2C, 3A

G.F.: 1B, 1C, 2A, 2B

I.E.‐A.: 1C, 2B

G.S.: 1C, 2B

M.M.: 1C, 2B

A.T: 1C, 2B

P.I.: 2B, 2C

S.C: 2C, 3B

G.M. 2C, 3B

P.B.: 2C, 3B

A.B: 1A, 1B, 2A, 2C, 3A, 3B

A.P.: 1A, 1B, 2A, 2C, 3A, 3B

## Supporting information


**Fig. S1** Tor1a^+/Δgag^ mice display an increased level of synaptotagmin I (Syt I) in the dorsal striatum. (**A**) Representative confocal images showing an increase in the Syt I fluorescence signal in the dorsal striata from Tor1a^+/Δgag^ mice (Tor1a^+/+^ = 1989 ± 537 μm^2^, N = 6; Tor1a^+/Δgag^ = 6947 ± 1356 μm^2^, N = 6; **P < 0.01). Scale bar = 10 μm. (**B**) Representative Western Blot (WB) showing Syt I protein level increase in the dorsal striata of Tor1a^+/Δgag^ mice when compared to Tor1a^+/+^ mice. The graph shows the quantitative analysis of Syt I levels normalized to Tor1a^*+/+*^ mice. The amount of Syt I was quantified relatively to β‐actin. Data are presented as mean ± SEM (Tor1a^+/+^ = 1 ± 0.11, N = 12; Tor1a^+/Δgag^ = 2.12 ± 0.32, N = 12; *P < 0.05).

## Data Availability

The data that support the findings of this study are available from the corresponding author upon reasonable request.
