# Supplementary figures and images for: Alpha‐Synuclein is Involved in DYT1 Dystonia Striatal Synaptic Dysfunction
Source: Mov Disord. 2022 Apr 14;37(5):949–61. doi: 10.1002/mds.29024 (PMC9323501; doi:10.1002/mds.29024)

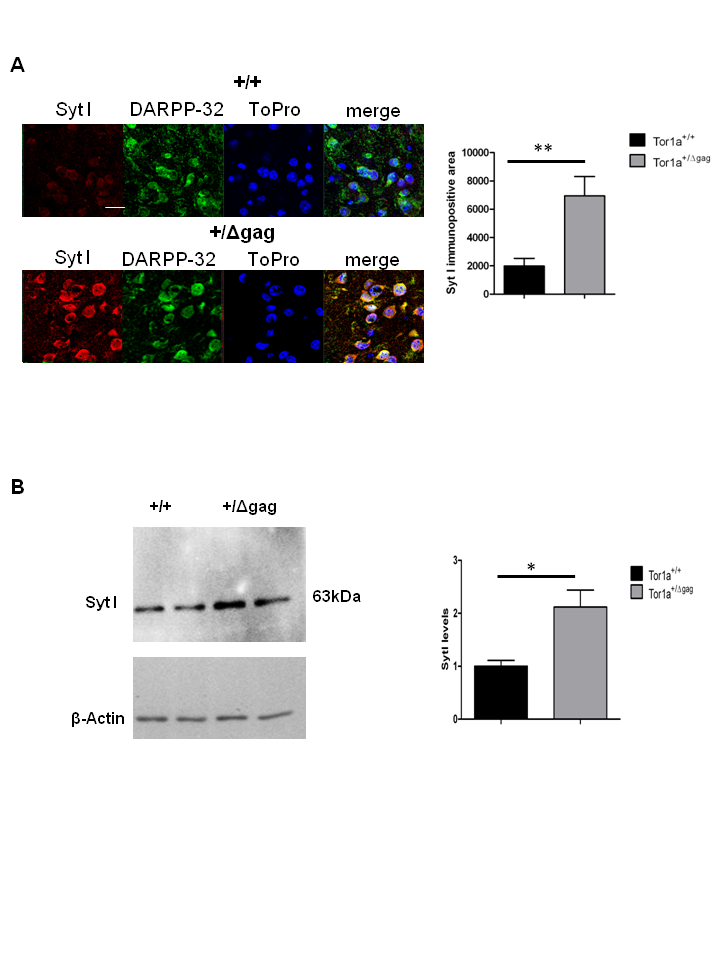

Supplement: Supplementary file 1 — Fig. S1 Tor1a+/Δgag mice display an increased level of synaptotagmin I (Syt I) in the dorsal striatum. (A) Representative confocal images showing an increase in the Syt I fluorescence signal in the dorsal striata from Tor1a+/Δgag mice (Tor1a+/+ = 1989 ± 537 μm2, N = 6; Tor1a+/Δgag = 6947 ± 1356 μm2, N = 6; **P < 0.01). Scale bar = 10 μm. (B) Representative Western Blot (WB) showing Syt I protein level increase in the dorsal striata of Tor1a+/Δgag mice when compared to Tor1a+/+ mice. The graph shows the quantitative analysis of Syt I levels normalized to Tor1a +/+ mice. The amount of Syt I was quantified relatively to β‐actin. Data are presented as mean ± SEM (Tor1a+/+ = 1 ± 0.11, N = 12; Tor1a+/Δgag = 2.12 ± 0.32, N = 12; *P < 0.05). [file MDS-37-949-s001.tif]
